# Supplementary material for: Baseline Characterization of the Gut Microbiota of Field and Colony Populations of Phlebotomus tobbi and Preliminary Assessment of the Anti-Leishmanial Activity of Cultivable Bacteria
Source: Pathogens. 2026 Jun 23;15(7):658. doi: 10.3390/pathogens15070658 (PMC13416292; doi:10.3390/pathogens15070658)
Supplement: Supplementary file 1 [file pathogens-15-00658-s001.zip › pathogens-4357168-File S1/Heatmap/7.species/heatmap_without_sample_clustering.pdf]

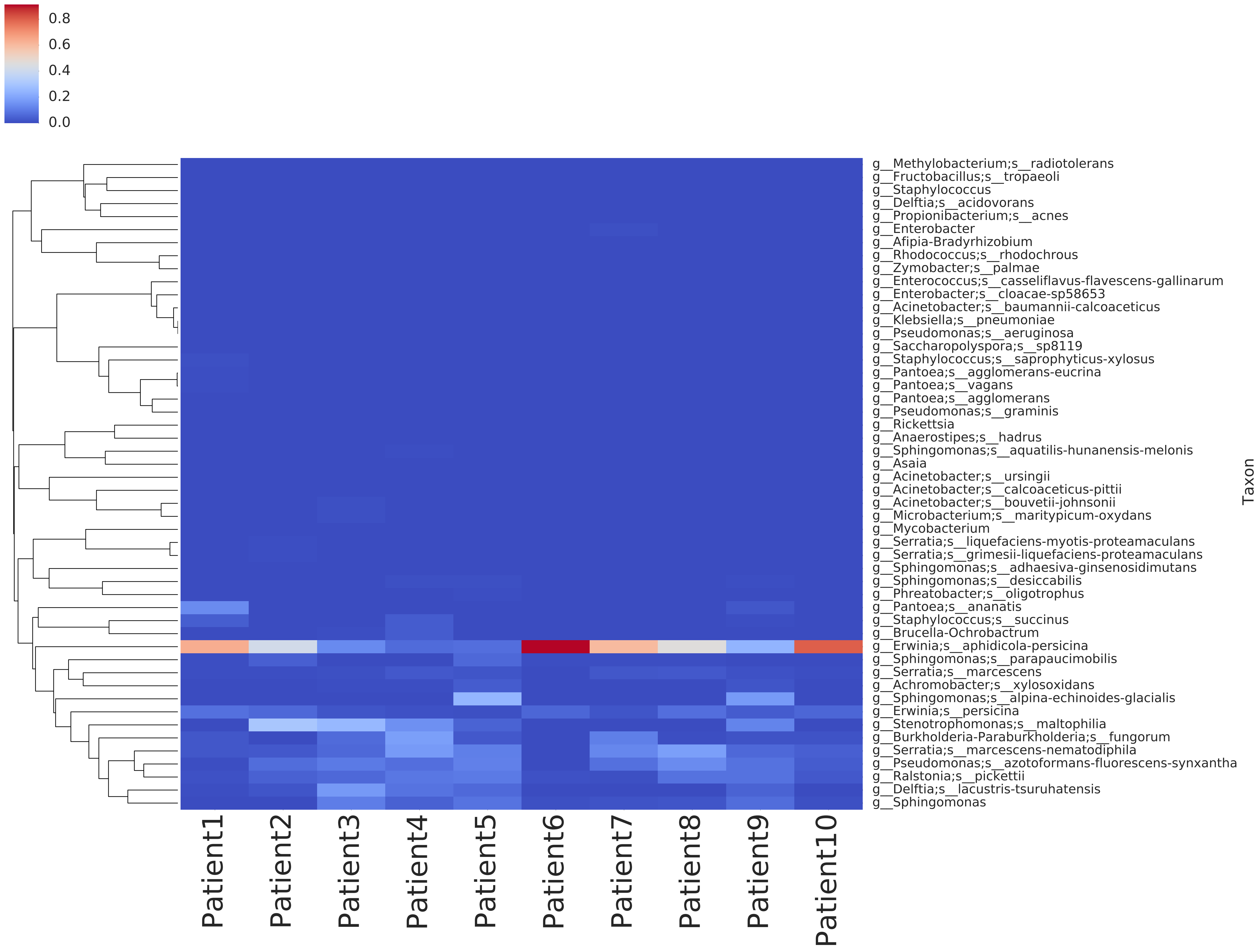
0.8
0.6
0.4
0.2
0.0
g__Methylobacterium;s__radiotolerans
g__Fructobacillus;s__tropaeoli
g__Staphylococcus
g__Delftia;s__acidovorans
g__Propionibacterium;s__acnes
g__Enterobacter
g__Afipia-Bradyrhizobium
g__Rhodococcus;s__rhodochrous
g__Zymobacter;s__palmae
g__Enterococcus;s__casseliflavus-flavescens-gallinarum
g__Enterobacter;s__cloacae-sp58653
g__Acinetobacter;s__baumannii-calcoaceticus
g__Klebsiella;s__pneumoniae
g__Pseudomonas;s__aeruginosa
g__Saccharopolyspora;s__sp8119
g__Staphylococcus;s__saprophyticus-xylosus
g__Pantoea;s__agglomerans-eucrina
g__Pantoea;s__vagans
g__Pantoea;s__agglomerans
g__Pseudomonas;s__graminis
g__Rickettsia
g__Anaerostipes;s__hadrus
g__Sphingomonas;s__aquatilis-hunanensis-melonis
g__Asaia
g__Acinetobacter;s__ursingii
g__Acinetobacter;s__calcoaceticus-pittii
g__Acinetobacter;s__bouvetii-johnsonii
g__Microbacterium;s__maritypicum-oxydans
g__Mycobacterium
g__Serratia;s__liquefaciens-myotis-proteamaculans
g__Serratia;s__grimesii-liquefaciens-proteamaculans
g__Sphingomonas;s__adhaesiva-ginsenosidimutans
g__Sphingomonas;s__desiccabilis
g__Phreatobacter;s__oligotrophus
g__Pantoea;s__ananatis
g__Staphylococcus;s__succinus
g__Brucella-Ochrobactrum
g__Erwinia;s__aphidicola-persicina
g__Sphingomonas;s__parapaucimobilis
g__Serratia;s__marcescens
g__Achromobacter;s__xylosoxidans
g__Sphingomonas;s__alpina-echinoides-glacialis
g__Erwinia;s__persicina
g__Stenotrophomonas;s__maltophilia
g__Burkholderia-Paraburkholderia;s__fungorum
g__Serratia;s__marcescens-nematodiphila
g__Pseudomonas;s__azotoformans-fluorescens-synxantha
g__Ralstonia;s__pickettii
g__Delftia;s__lacustris-tsuruhatensis
g__Sphingomonas
Taxon
Patient1
Patient2
Patient3
Patient4
Patient5
Patient6
Patient7
Patient8
Patient9
Patient10
